# Comparison of Stress-Impedance Effect in Amorphous Ribbons with Positive and Negative Magnetostriction

**DOI:** 10.3390/ma12020275

**Published:** 2019-01-16

**Authors:** Piotr Gazda, Michał Nowicki, Roman Szewczyk

**Affiliations:** Warsaw University of Technology, Institute of Metrology and Biomedical Engineering, 02-525 Warsaw, Poland; nowicki@mchtr.pw.edu.pl (M.N.); szewczyk@mchtr.pw.edu.pl (R.S.)

**Keywords:** metallic glass, stress-impedance, Villari effect, strain sensing

## Abstract

The SI (stress-impedance) effect in amorphous ribbons with varying magnetostriction was investigated. Iron- and cobalt-based ribbons with different magnetostriction coefficients were put under tensile stress in a dead weight tester and the impedance change was investigated in function of applied stresses. Significant differences of characteristics are presented. Stress-impedance analog of Villari reversal point was observed. The reversal point showed driving current frequency dependence, in which this point manifests for different stress values. Based on the obtained SI characteristics and magnetoelastic hysteresis, the most appropriate stress-sensing material was selected for development of precise small forces sensor.

## 1. Introduction

Since the discovery of the Giant Magneto-Impedance (GMI) effect in 1994 [1,2] intensive works are focused on research of this effect in amorphous soft magnetic wires and ribbons, due to their potential applications in magnetic field sensing and measurement of the phenomena associated with it. The GMI effect consists of a significant (gigantic) change in the impedance of the high frequency AC (alternative current) current conductor under the influence of a constant external magnetic field *H*. The GMI effect can be understood in the context of classical electrodynamics, connecting the change of penetration depth *δ* with a DC (direct current) axial magnetic field [3]. Novel amorphous soft magnetic materials exhibit large magnetic permeability, which changes significantly under the influence of a relatively small magnetic field. Change in permeability directly influences the penetration depth, which in turn affects the conductor’s impedance. 

The change of magnetic permeability can be also influenced by stresses in the material. The effect associated with a significant change in the impedance of the conductor under the stress is called the stress impedance (SI). This effect was first described by Shen [4] et al. with impedance change of 14% for negative magnetostriction, amorphous wire, with a 20 MHz driving signal and tensile stresses of 14 MPa. Later, the effect was used to develop stress sensors based on amorphous wire [5,6,7]. Since then, SI effect has been studied in thin layers [8,9,10], in sandwiched structures [11,12,13,14,15], as well as in amorphous ribbons [16,17,18,19,20] and wires [21,22,23].

Studies on the effect of stresses on magnetic permeability are associated with the Villari effect. It relates the deformation of the material structure with a change in the magnetic state of the material. It can, for example, be represented as a change in magnetic induction B for a constant field H under the influence of stress [24]. Although this effect was observed in the mid-nineteenth century [25], it is still the object of interest of many scientists with regard to the new class of magnetic materials [26].

In the presented work, comparative stress-impedance measurements in amorphous ribbons with different values and sign of magnetostriction are described. It was expected that significant SI effect will be observed both for magnetostrictive, as well as near-zero magnetostriction ribbons. Basing on obtained experimental results, the paper presents the similarity between the non-monotonous shape of the SI curve with the magnetoelastic Villari effect. The Stress-Impedance Villari point dependence on the frequency of the driving signal is depicted as well. Furthermore, analysis of obtained SI coefficients, and hysteresis of the obtained characteristics suggest the most promising material for stress-sensing applications.

## 2. Materials and Methods

During the investigation Fe-based, Fe-Ni and Co-based amorphous ribbons in as-cast state were used. Table 1 summarizes the properties of the samples. Ribbons were cut into samples of width *w* equal 1 ± 0.1 mm and lengths *l* equal 50 mm.

The impedance *Z* of the ribbon was measured by four-probe method using LCR Bridge (Microtest 6630E, New Taipei City, Taiwan). Research was carried out for low and medium impedance test frequencies (<5 MHz) with a driving current *I_rms_* equal 10 mA. Special program for controlling measurement system and collecting measurement data was developed in National Instruments LabVIEW environment (v.17, National Instruments, Austin, TX, USA).

The SI ratio is calculated in respect to the minimal applied stress *σ*, using following formula:

(1) $$\frac{\Delta Z}{Z}(\%)=100\cdot \frac{Z(\sigma )-Z(\sigma_{min})}{Z(\sigma_{min})}$$

**Figure 1:**
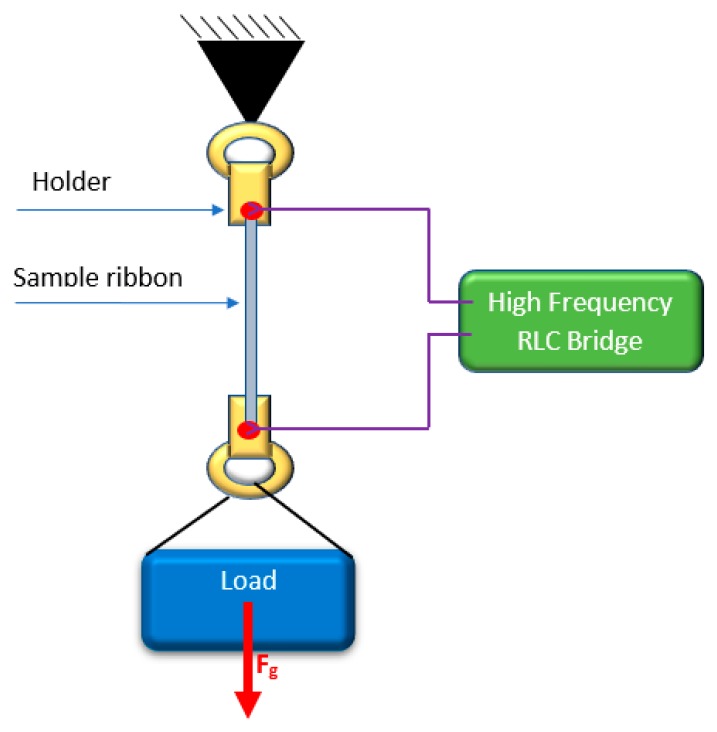
Schematic diagram of the developed SI measurement system.

The tensile stress of up to about 200 MPa was applied by the attached loads. The minimal stress *σ_min_* was about 3 MPa due to the weight of the holder to which the sample was attached. Figure 1 presents the schematic diagram of the measurement test stand and illustrates the method of applying the stresses. The loads were set in a cycle from maximal to minimal value with constant increment of 24.80 g, and from minimal to maximal in the next step to determine the measurement hysteresis. The hysteresis error e_h_ is calculated using following formula:

(2) $$e_{h}(\%)=100\cdot \frac{{\left(Z_{d}-Z_{u}\right)}_{max}}{Z_{max}-Z_{min}}$$

where (*Z_d_* − *Z_u_*)*_max_*—maximum hysteresis loop width, *Z_max_*—maximal indication obtained during hysteresis measurement, *x_min_*—the minimal display obtained when measuring hysteresis.

In order to accurately determine the stress in the given measurement point, measurements of the weight of loads, and precise measurement of the width of the sample were carried out. An analytical scale (XA 82/220 3Y, Radwag, Radom, Poland) was used to measure the set load. Measurement of the width was made with the help of a digital optical microscope (VMS-004D, Veho, Hampshire, UK). The gravity constant used for determining stresses g equal 9.81229 m·s^−2^ was taken from [31]. The investigated sample was placed in a permanent magnetic field. Three pairs of perpendicularly set Helmholtz coils, supplied by three separated DC Power Supplies, with the application of a magnetoresistive sensor HMR2000 (Honeywell, Phoenix, AZ, USA) as feedback, were used to produce stable DC magnetic field of 40 A/m along the axis of the test sample—typical value of Earth magnetic field present in Europe. The reason for the study in such conditions was, on the one hand, the elimination of the influence on impedance of external magnetic field and, on the other hand, measurements in an environment corresponding to the magnetoelastic sensors working conditions—measurements in presence of Earth stable DC magnetic field. The measurements of impedance were performed at room temperature. 

## 3. Results

### 3.1. SI Measurments

**Figure 2:**
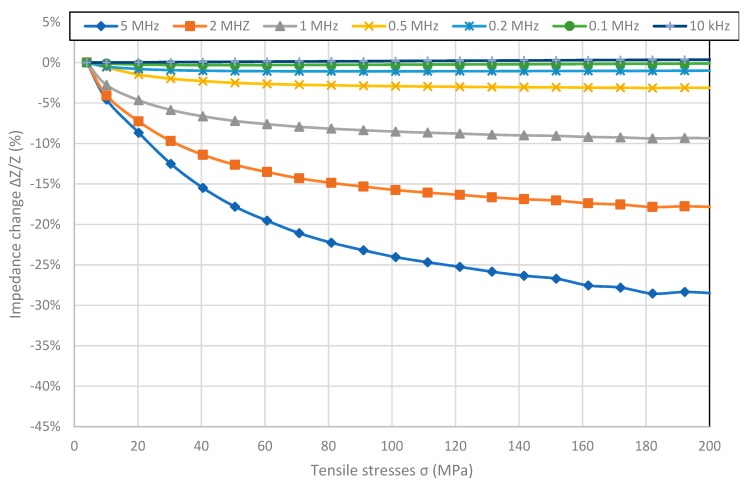
The tensile stress *σ* dependence of impedance *Z* in Fe_80_B_11_Si_9_ sample.

**Figure 3:**
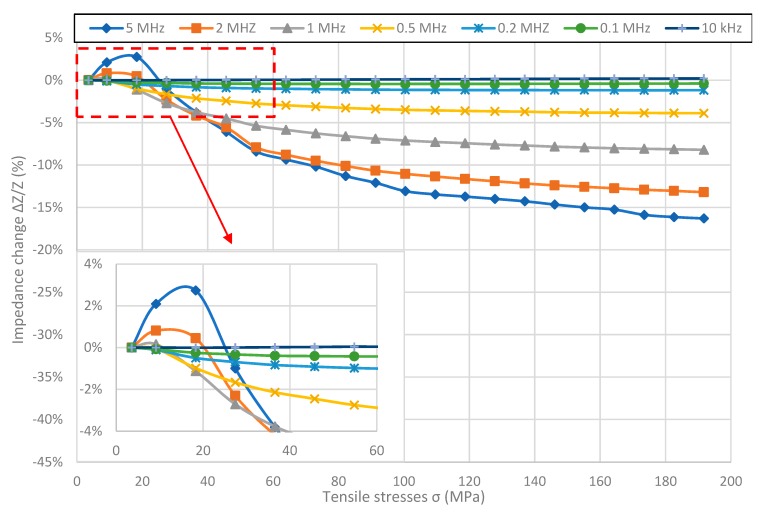
Dependence of sample Fe_40_Ni_38_Mo_4_B_18_ impedance on tensile stress *σ*.

**Figure 4:**
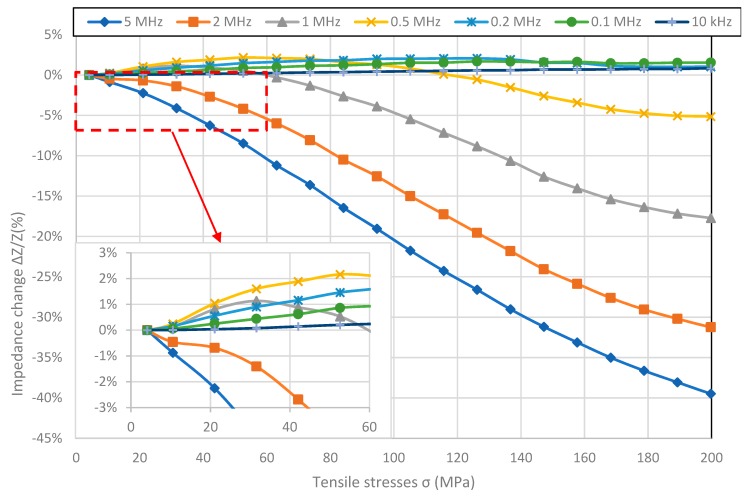
Dependence of sample Co_70_Fe_5_Ni_2_Mo_5_B_3_Si_15_ impedance on tensile stress *σ*.

**Figure 5:**
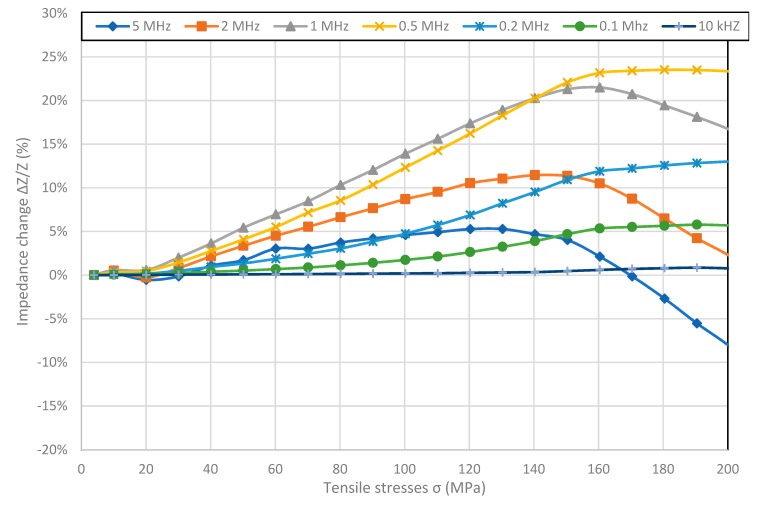
Dependence of sample Co_84_Fe_1.5_Mo_2_Mn_1.5_Si_7_B_2_ impedance on tensile stress *σ*.

Figure 2, Figure 3, Figure 4 and Figure 5 present the longitudinal tensile stress dependence of the SI for amorphous ribbons. For all of the investigated ribbons, a change in impedance under the influence of applied stresses was observed. For all of them—excluding Co_71_Fe_1_Mo_1_Mn_4_Si_14_B_9_—the SI effect rises with the rise of frequency. The comparison of graphs shows significant difference in SI character. 

Figure 2 shows SI for Fe_80_B_11_Si_9_ ribbon with high magnetostriction saturation *λ_s_*value. The max SI value equal to −29.07% was reported for 5 MHz frequency. For all of the frequencies the maximal value of Impedance *Z* is for the minimal stresses and graphs exhibit saturation for higher stress values. The value of stress for which the saturation of the SI effect is achieved, increases with frequency and was not reached for frequencies above 1 MHz.

For the Fe_40_Ni_38_Mo_4_B_18_ sample (Figure 3), shape of the graph changes with the frequency. For frequencies below 1 MHz, graphs are hyperbolic like for Fe_80_B_11_Si_9_ ribbon. For 1 MHz and higher frequencies graphs aren’t monotonic and have pronounced reversal point. What is more, the stress value for which the inflection point occurs, increases with frequency, as can be seen more clearly at the inside of Figure 3. The maximal SI value was obtained for the 5 MHz frequency and was absolutely 16.30%, while the difference between the maximal and minimal impedance value was 19%. Similarly to Fe_80_B_11_Si_9_ samples, the SI effect for given stresses reaches saturation for frequencies below 1 MHz, and is close to saturation for higher frequencies.

In the Figure 4, the results of measurements of Co_70_Fe_5_Ni_2_Mo_5_B_3_Si_15_ sample are presented. SI values are by far the largest of the measured samples and are −41.73% for 5 MHz. The effect in the entire measured range of stresses is not saturated and the nature of the occurrence of the reversal point is different than for the other samples. The value of stresses for which the reversal point occurs decreases with frequency, and for frequencies above 1 MHz does not occur. SI values are negative for frequencies above 0.2 MHz, and positive for lower frequencies. 

Figure 5 shows results of measured stress impedance effect for nearly zero magnetostriction Co_84_Fe_1.5_Mo_2_Mn_1.5_Si_7_B_2_ sample. Unlike other samples, SI values in a wide range of stresses are positive for all frequencies. The reversal point occurs for frequencies above 0.5 MHz and the stress value for which it occurs increases with frequency. In the measured range, the highest SI value of 23.52% was achieved for the frequency of 0.5 MHz. Because frequencies above 1MHz did not reach saturation in the case of extended measurements, they could reach a higher value of max-min SI.

### 3.2. Hysteresis of Stress Measurments

**Figure 6:**
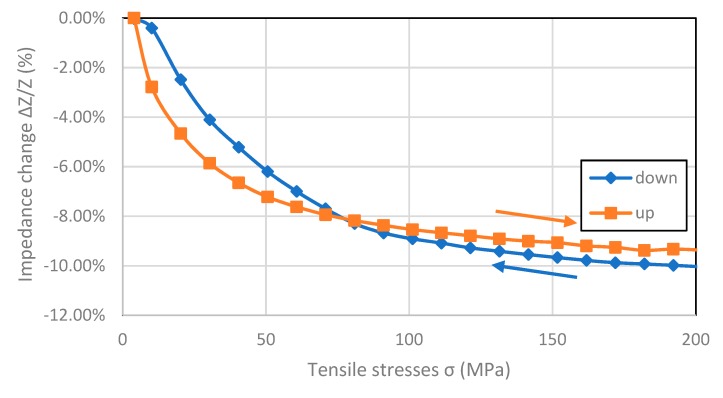
Hysteresis SI measurements of Fe_80_B_11_Si_9_ sample (for 1 MHz driving signal).

**Figure 7:**
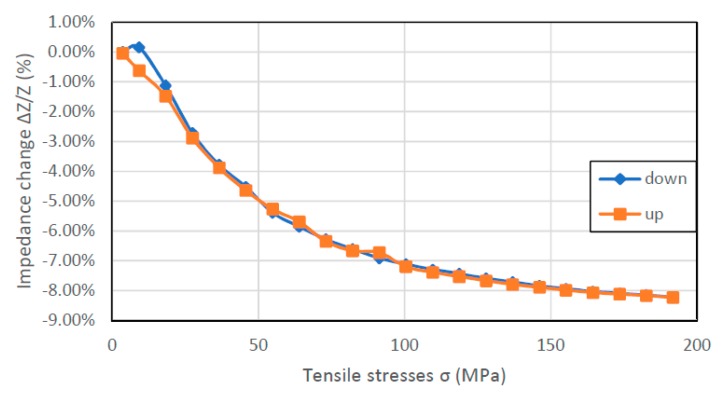
Hysteresis SI measurements of Fe_40_Ni_38_Mo_4_B_18_ sample (for 1 MHz driving signal).

**Figure 8:**
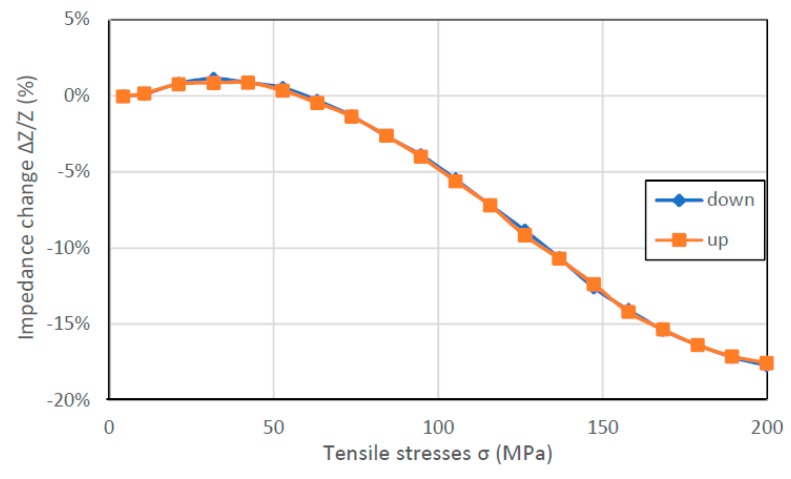
Hysteresis SI measurements of Co_70_Fe_5_Ni_2_Mo_5_B_3_Si_15_ sample (for 1 MHz driving signal).

**Figure 9:**
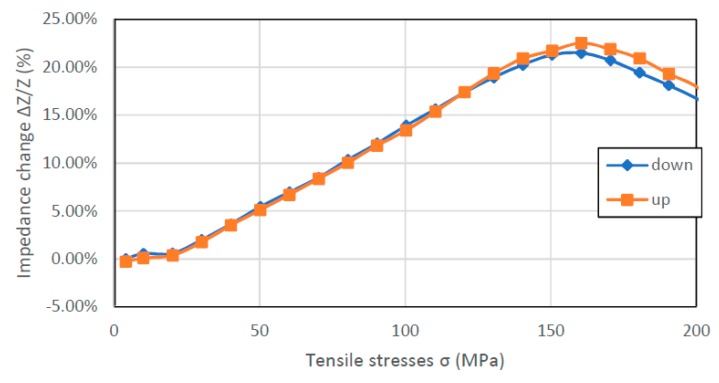
Hysteresis SI measurements of Co_84_Fe_1.5_Mo_2_Mn_1.5_Si_7_B_2_ sample (for 1 MHz driving signal).

In Figure 6, Figure 7, Figure 8 and Figure 9, the results of the hysteresis of the indications of the measured samples at 1 MHz are presented. The least hysteretic properties have a Co_70_Fe_5_Ni_2_Mo_5_B_3_Si_15_ sample (Figure 8)—the hysteresis error value was 1.75%, which is important from application point of view. The worst sample in this regard turned out to be Fe_80_B_11_Si_9_ ribbon—the hysteresis error was 23.5%. Fe_40_Ni_38_Mo_4_B_18_ and Co_71_Fe_1_Mo_1_Mn_4_Si_14_B_9_ samples have unambiguous sections. For the Fe_40_Ni_38_Mo_4_B_18_ ribbon useful range is 25–200 MPa and for Co_71_Fe_1_Mo_1_Mn_4_Si_14_B_9_ ribbon the 0–125 MPa region. For these, the hysteresis error value was 9.43% and 6.54%, respectively.

## 4. Discussion

The dependence binding the ribbon impedance *Z* to the penetration depth *δ* is obtained by inserting the solution of the Maxwell equation into an equation describing the impedance using the surface impedance tensor (the transformation is described in detail in [3,32]):

(3) $$Z=R_{dc}\cdot i\cdot \left(1+i\right)\cdot \delta \cdot a\cdot \cot h\left(i\cdot \left(1+i\right)\cdot \delta \cdot a\right)$$

where *R_dc_* is the dc electrical resistance, *i* is imaginary unit and *2a* is the thickness of ribbon. The function for the penetration depth *δ* is expressed as follows:

(4) $$\delta =\frac{c}{\sqrt{4\cdot \pi^{2}\cdot f\cdot \kappa \cdot \mu_{0}\cdot \mu_{r}}}$$

where *c* is the speed of light, *f* is the frequency of the AC current passed along the sample, *κ* is the electrical conductivity, *μ*_0_ is magnetic permeability of the vacuum and *μ_r_* is relative permeability. 

Equations (3) and (4) indicate that the change in the impedance of the conductor is the result of change of its permeability. It is known that magnetic permeability strongly depends on the magnetic field acting on it [33]. However, the complete quantitative model of permeability changes in the function of mechanical stresses *σ* was still not presented. The most advanced solution was proposed by Sablik et al. In this solution the effective field *H_eff_* acting on the sample considers also the influence of mechanical stresses. Using the Jiles–Sablik model, *H_eff_* can be expressed as [34]:

(5) $$\vec{H_{eff}}=\vec{H}+\alpha M+\vec{H_{\alpha }}$$

The individual components are defined as: *H* is the sum of exciting AC field and applied DC bias field, *α* is a dimensionless mean field representing interdomain coupling and *M* is the magnetization. The component of the magnetizing field connected with mechanical stress-induced anisotropy field is given as [34]:

(6) $$\vec{H_{\alpha }}=\frac{3\lambda_{s}\sigma }{2\mu_{0}M_{s}}\frac{M}{M_{s}}$$

where *M_s_* is the saturation magnetization. However, this model can only be used for the small mechanical stress values. In the range of high stresses that occurred in this study, saturation magnetostriction is not constant. Moreover, it was observed that for higher values of mechanical stresses, saturation magnetostriction can change its sign [35]. 

In the experiment, the composition *H* was constant and did not affect the impedance change. Comparing the measurements, it can be noticed that the scale of the SI effect depends primarily on magnetic permeability and secondly on magnetostriction. The largest SI ratio was obtained for a ribbon with a nearly zero magnetostriction but with a very high permeability, and the lowest for Fe-Ni alloy which had much lower permeability than Co-based ribbon, and much smaller magnetostriction than the Fe-based one. 

Large impedance changes for the sample with almost zero magnetostriction, due to the expected significant change in saturation magnetostriction under the influence of stresses. The influence of stresses on the magnetostriction of amorphous alloys with a negligible value of saturation magnetostriction factor has not been sufficiently studied; however, previous research confirms these phenomena [36].

The hysteresis of measurements visible in Figure 6 and Figure 9 can be explained by magnetoelastic hysteresis. External stresses interact with internal stresses, causing residual stresses and the sample does not return to its original state. This effect can be observed in the whole range of stresses or only in certain intervals.

## 5. Conclusions

The novelty related to the SI effect is the occurrence of a turning point for some characteristics. Such a turning point is characteristic of the Villari effect [37]. We propose this phenomenon to be named shortly as SI-Villari point. It was also observed that the turning point stress values shifted along with the frequency. The significance of the obtained results is that as the frequency increases, the SI-Villari point will shift towards lower stresses for positive magnetostriction and towards higher stresses for negative magnetostriction. In order to better understand the phenomenon, further research is needed. What is more, also near-zero magnetostriction ribbon exhibits significant SI effect, despite the traditional connection of direct and inverse magnetoelastic effects.

The performed measurements indicate that the best investigated material for the development of a stress sensor based on the SI effect is a Co_70_Fe_5_Ni_2_Mo_5_B_3_Si_15_ ribbon, which shows the highest SI factor and very low hysteresis of indications. The range useful for application is between 80 and 160 MPa with monotonic and nearly linear characteristics.

## Figures and Tables

**Table 1:** A summary of the essential properties of the compared amorphous ribbons [27,28,29,30].

Manufacturer and Trade Name	Chemical Composition	Thick-ness *t* (µm)	Maximal Permeability in As-Cast State *μ*	Magneto-Striction in Saturation *λ*_s_ (μm/m)	Saturation Induction *B_s_* (T)	Coercivity *H_c_* (A/m)
Metglas SA1	Fe_80_B_11_Si_9_	23	45,000	27	1.56	1.05
Metglas 2826MB	Fe_40_Ni_38_Mo_4_B_18_	29	>50,000	12	0.88	3.08
Metglas 2705M	Co_70_Fe_5_Ni_2_Mo_5_B_3_Si_15_	22	290,000	<0.5	0.77	0.95
Vacuum-schmelze 6030 D30	Co_84_Fe_1.5_Mo_2_Mn_1.5_Si_7_B_2_	21	450,000	−11.8	0.82	2.03
